# Maternal anxiety and child emotional distress during war: the buffering roles of maternal self-efficacy and parent–child communication

**DOI:** 10.3389/fpsyg.2026.1641327

**Published:** 2026-02-04

**Authors:** Ortal Buchnik-Atzil, Tzlil Einziger, Yarden Gliksman, Lilac Lev-Ari

**Affiliations:** Department of Behavioral Sciences, Ruppin Academic Center, Emek Hefer, Israel

**Keywords:** child emotional distress, maternal anxiety, maternal self-efficacy, parent–child communication, protective factors, war-related stress

## Abstract

**Introduction:**

War-related stressors can have profound effects on family psychological wellbeing, with young children being particularly vulnerable to emotional distress when their parents experience heightened anxiety. This study examined the association between maternal anxiety and child emotional distress during a period of war-related stress and explored parental factors that may protect children from heightened distress.

**Methods:**

The sample included 135 mothers and their children residing in central Israel, who were exposed to daily missile attacks during the first month of the “Iron Swords” war. Mothers completed standardized questionnaires assessing maternal anxiety (GAD-7), parental self-efficacy (MaaPS-SF), war-related parent–child communication, and child emotional distress (PEDS).

**Results:**

Results indicated that higher levels of maternal anxiety were positively associated with greater child emotional distress. However, this association was not significant among mothers with higher self-efficacy, nor among those who engaged in conversations with their children about the war. Maternal anxiety increased with child age and decreased with maternal self-efficacy, yet the buffering roles of self-efficacy and parent–child conversations were consistent for children of all ages.

**Discussion:**

These findings emphasize the importance of supporting parental self-efficacy and fostering open parent– child communication as protective factors that may enhance family resilience during wartime.

## Introduction

On October 7th, 2023, Israel came under an unprecedented terror attack that included brutal atrocities and was followed by extensive rocket fire across the country (Levany et al., 2023). This massive terrorist attack and the subsequent “Iron Sword” war exposed Israeli citizens to extreme stress. The beginning of the war was marked by a sudden transition from civilian routine to an acute security threat. This shift affected not only border regions but also areas geographically distant from them, which under routine conditions prior to the war, are not typically exposed to air-raid sirens. Families with young children may be particularly vulnerable during major life stressors, as they must navigate both their own responses to the crisis and their children’s needs for security and stability (Bradley, 2007; Kostenko et al., 2024; Rizzi et al., 2022; Walsh, 1996).

Research consistently shows that exposure to terrorism and war can significantly impact children’s mental health, leading to increased symptoms of post-traumatic stress disorder (PTSD), anxiety, and depression (Bitton and Laufer, 2018; Slone and Mann, 2016). Children may experience a range of emotional and behavioral difficulties, including increased fears, social problems, and aggressive behaviors (Braun-Lewensohn et al., 2009; Thabet et al., 2016). Importantly, these effects are not limited to direct exposure; studies following events such as 9/11 demonstrated significant emotional impact even on children who were not directly exposed to the attacks (Kinzie et al., 2002; Otto et al., 2007).

While war-related stressors may affect the entire family system, young children rely heavily on caregivers for co-regulation of emotion (Silkenbeumer et al., 2016). In contexts of exposure to war and terrorism, characterized by uncertainty and extreme stress, maternal anxiety may therefore be particularly important for children’s adjustment. Indeed, maternal anxiety has consistently been identified as a predictor of child emotional distress (Creswell et al., 2008; Thakar et al., 2013; Zamir et al., 2020); through intergenerational transmission (Cohen, 2019). For example, mothers experiencing elevated anxiety levels during crisis periods showed impaired emotional regulation during parent–child interactions, leading to decreased empathetic responses to child distress (Bar-Sella et al., 2023). In addition, maternal anxiety symptoms during wartime can significantly impact children’s long-term emotional adjustment (Slone and Mann, 2016; Tangir et al., 2017). Mothers experiencing increased anxiety may demonstrate reduced tolerance for their children’s negative emotions, potentially interfering with positive mother–child interactions and compromising emotional co-regulation processes (Creswell et al., 2008; Morris, 2015).

Identifying protective factors that may buffer the adverse effects of maternal anxiety on child emotional distress is therefore essential. These insights can inform targeted interventions to support both maternal and child well-being during stressful periods. One such factor is parental self-efficacy, defined as parents’ beliefs about their ability to parent effectively and to influence their child’s health and positive developmental outcomes (Albanese et al., 2019). High parental self-efficacy has been consistently linked to the well-being of both parents and children (Albanese et al., 2019; Jones and Prinz, 2005), and to more adaptive parenting behaviors (e.g., higher sensitivity and emotional availability (Albanese et al., 2019). Similarly, maternal confidence and competence were found to be associated with less parenting stress (Liu et al., 2012). From a broader theoretical perspective, Bandura’s framework conceptualizes self-efficacy as a key determinant of how individuals manage emotional responses, confront demanding situations, and sustain engagement under stress. Individuals who perceive themselves as capable are more likely to invest effort, persist despite difficulties, and respond to stress in a more adaptive manner (Bandura, 1997).

In high-stress contexts (e.g., during wartime), parental self-efficacy may therefore function as a protective factor by supporting parents’ capacity to remain consistent and responsive in their caregiving. Previous research examined physiological stress responses in mothers of young adolescents during a highly stressful laboratory task. Mothers with higher parental self-efficacy showed lower stress and anxiety while observing their children face challenges. These findings suggest that greater maternal self-efficacy is associated with reduced physiological stress, enabling mothers to remain more composed and supportive and to provide more effective assistance during stressful situations (Buchanan et al., 2022).

Another potential protective factor buffering the risk posed by maternal anxiety is parents’ ability to communicate with their children about war-related events. Evidence suggests that parental avoidance of discussing traumatic events can worsen a child’s symptoms (see review of (Afzal et al., 2023; Sloover et al., 2024). Parents who can engage in such conversations while managing their own anxiety may be particularly effective in supporting their children’s adjustment during times of stress. However, research examining parents’ ability to discuss and explain their children’s stressful experiences remains limited.

Despite growing research on the psychological impact of war on children and parents (Bitton and Laufer, 2018; Slone and Mann, 2016), several important gaps remain. First, relatively little is known about young children’s emotional adjustment under conditions of ongoing threat. Second, the potential buffering role of maternal self-efficacy and war-related parent–child communication in high-stress contexts such as wartime has received limited empirical attention. The current study addresses these gaps by examining these interrelated processes among mothers and young children exposed to continuous war-related stress.

## The present study

The current study examines the associations between maternal anxiety and child emotional distress in Israeli families following the October 7th attacks and during the subsequent war period. Specifically, we investigate whether maternal self-efficacy and war-related conversations with children can moderate the relation between maternal anxiety and child distress during war times. We conducted a survey 3 weeks after the onset of the ‘Iron Swords’ war, focusing on families on the home front who experienced daily rocket attacks. We aimed to document anxiety levels among mothers and their children, to examine the associations between maternal anxiety and child emotional distress, and to investigate whether maternal self-efficacy and war-related conversations would serve as moderating protective factors, mitigating the risk posed by maternal anxiety. We hypothesized that while maternal anxiety would generally predict higher child distress, this relation would be weaker among mothers with high self-efficacy and among those who engaged in war-related conversations with their children.

## Method

### Participants

A sample of 135 mothers and children was collected 3 weeks after the beginning of the “Iron Swords” war (November 1–11, 2023), during a period characterized by a transition from civilian routine to acute security threat. All participants were part of the home front, living in the Sharon and Central regions of Israel, and were not directly exposed to the October 7th massacre. At this time-point, participants experienced air raid sirens and rocket fire and some of them had a first- or second-degree acquaintance who was harmed. However, none of the participants were physically injured, and their homes were not damaged. Note that these regions are geographically distant from the border regions, and during times of routine (i.e., before the beginning of the war), are not typically exposed to air-raid sirens.

The current study is part of a larger investigation examining emotional distress among children in Israel during the Iron Swords War. A total of 435 mothers initiated the survey, of whom 293 completed questionnaires regarding a child under 18 years of age. The inclusion criterion for the present study was being a mother of a child aged 2–8 years. Mothers of children outside this age range completed other age-appropriate questionnaires and were therefore not included in the present analyses. The final sample comprised 135 mothers reporting on children aged 2–8 years who completed the child primary outcome measure, the Pediatric Emotional Distress Scale (PEDS; Saylor et al., 1999), which is designed for this age range.

The mothers’ ages ranged from 26 to 51 years (*M* = 36.87, *SD* = 4.61). The children (53.3% female) were between 2 and 8 years old (*M* = 4.63, *SD* = 1.82). Socioeconomic status (SES), as reported by Israel’s Central Bureau of Statistics (CBS), indicated that 85% of the sample were classified as average or above. Regarding education, 92% of the sample held a bachelor’s degree or higher. In terms of marital status, 94% of the mothers were married, while the remaining 6% were widowed, divorced, or single mothers.

## Measures

*A General Questionnaire*. Mothers completed a general questionnaire consisting of four parts.

*A Demographic Questionnaire*. The first part included a demographic questionnaire that included information on family income, education, religious affiliation and level, number of children, area of residence, and other relevant details. Socioeconomic status (SES) was calculated as the average of standardized scores of family income and maternal education.

*Previous Potential Traumatic Events*. The second part contained a series of yes/no questions about exposure to potentially traumatic events (e.g., accidents, terrorist attacks) or other negative experiences (e.g., the death of a close person, divorce), as well as a history of mental health illness.

*War-Related Conversations*. In the third part, mothers reported on their communication with their children about the war, including whether they explained the situation to them and engaged in war-related conversations. This measure consisted of a single dichotomous item (i.e., yes/no) that indicated the presence or absence of war-related communication with the child. However, it did not capture other aspects of these conversations, such as frequency, content, or quality.

*War-Related Exposure.* In the final part, participants completed a short yes/no checklist regarding direct or indirect exposure to various aspects of the war (e.g., house damage, injury to themselves or others in their close or extended circles).

### Maternal measures

*Maternal Anxiety*. Maternal anxiety was assessed using the Generalized Anxiety Disorder 7-Item Scale (GAD-7; Löwe et al., 2008). The GAD-7 includes 7 items; mothers were asked to rate each item based on how often they had been bothered by various problems over the past 2 weeks (e.g., *not being able to stop or control worrying*), using a scale from 0 (*not at all*) to 3 (*nearly every day*). Cronbach’s alpha was 0.87.

*Maternal Self-Efficacy*. Self-efficacy was assessed using the Me as a Parent Scale-Short Form (MaaPS-SF; Matthews et al., 2022). The MaaPS-SF includes 4 items; mothers were asked to rate each item based on their perceptions of their parenting capabilities (e.g., *I have confidence in myself as a parent*) on a scale of 1 (*strongly disagree*) to 5 (*strongly agree*). Cronbach’s alpha was 0.82.

### Child outcomes

*Child Emotional Distress*. Child emotional distress was assessed using the Pediatric Emotional Distress Scale (PEDS; Saylor et al., 1999). The PEDS includes 17 items; for each item, mothers were asked to report certain behaviors exhibited by their child in the last few weeks (e.g., *refuse to sleep alone*), on a scale of 1 (*almost never*) to 4 (*very often*). The PEDS includes three subscales: Anxious/Avoidant (Cronbach alpha = 0.77), Fearful (Cronbach alpha = 0.78), and Acting Out (Cronbach alpha = 0.73), which comprise a total score, with higher values indicating more severe emotional distress. The total score showed good internal consistency (Cronbach’s alpha = 0.84) and has an established clinical cutoff score of 28.

### Procedure

Participants were recruited online via advertisements in social networks and participated in the study voluntarily. Participants were provided with a link to the online questionnaire, which was constructed using Qualtrics software. We conducted a lottery among all participants who filled out the entire survey, and five participants received vouchers of 100 NIS (about 25$). Informants were provided with a recruitment letter outlining the purpose of the study and the researchers’ contact information. The participants were assured of anonymity, confidentiality, and their right to withdraw from the study at any time, and that they could keep answering the questionnaire for up to a week. Participants who agreed to participate were required to sign an informed consent form. The ethics committee at the Ruppin Academic Center approved the study.

### Data analytic approach

Moderation analyses were conducted using hierarchical multiple regression models. Family SES, child age, and child gender, as well as the main effects of maternal anxiety, maternal self-efficacy, and war-related conversations, were entered at Step 1. Interaction terms between maternal anxiety and war-related conversations and between maternal anxiety and maternal self-efficacy were entered at Step 2. For significant interactions, conditional effects of maternal anxiety on child emotional distress were estimated at specific values of the moderators (maternal self-efficacy at ± 1 SD from the mean; war-related conversations coded as presence vs. absence).

## Results

### Preliminary analyses

We first examined the correlation between the study variables and background variables (e.g., SES, child age, and gender). Child age was positively correlated with maternal anxiety and negatively correlated with maternal self-efficacy. Specifically, older ages were associated with higher maternal anxiety and lower maternal self-efficacy. Child emotional distress was higher among girls (*M* = 34.61, *SD* = 9.07) compared to boys (*M* = 30.48, *SD* = 7.01), *t*(133) = −2.93, *p* < 0.01. SES was marginally significantly and negatively correlated with child emotional distress, *r* = −0.16, *p* < 0.10. See Table 1. Therefore, we included these background variables as covariates in the regression analysis. Exposure to previous potential traumatic events or other negative adverse experiences, as well as a history of mental health illness, was not related to maternal anxiety.

**Table 1:** Descriptive statistics and intercorrelation among study variables.

Variable	*M*	*SD*	1	2	3	4	5	6
1. SES	3.50	0.72						
2. Child gender			−0.19*					
			[−0.35, −0.02]					
3. Child age	4.63	1.82	0.08	−0.06				
			[−0.09, 0.24]	[−0.23, 0.11]				
4. Maternal anxiety	10.58	4.70	−0.09	0.16	0.20*			
			[−0.26, 0.08]	[−0.01, 0.32]	[0.03, 0.35]			
5. Maternal self-efficacy	15.31	2.47	0.11	−0.12	−0.26**	−0.23**		
			[−0.07, 0.27]	[−0.28, 0.05]	[−0.42, −0.10]	[−0.38, −0.06]		
6. War-related conversation			0.12	−0.13	0.56**	0.05	−0.18*	
			[−0.05, 0.29]	[−0.29, 0.04]	[0.43, 0.66]	[−0.12, 0.22]	[−0.34, −0.01]	
7. Child emotional distress	32.68	8.41	−0.17	0.25**	0.04	0.34**	−0.29**	0.08
			[−0.33, 0.00]	[0.08, 0.40]	[−0.13, 0.21]	[0.19, 0.48]	[−0.44, −0.13]	[−0.09, 0.25]

M and SD are used to represent mean and standard deviation, respectively. Values in square brackets indicate the 95% confidence interval for each correlation. * indicates *p* < 0.05. ** indicates *p* < 0.01. SES = socio-economic status. War-related conversations were coded as 0 = absence of conversations and 1 = presence of conversations.

Table 1 presents the descriptive statistics and intercorrelation among study variables of interest. Maternal anxiety was negatively correlated with maternal self-efficacy, and positively with child emotional distress. Maternal self-efficacy was negatively correlated with war-related conversation and child emotional distress.

### Anxiety levels among mothers and children

Nearly half of the mothers (49%) scored above the clinical cutoff on the GAD-7 (≥10), indicating clinically significant anxiety symptoms. Among the children, 65% scored above the clinical cutoff on the PEDS (≥28), suggesting elevated distress levels.

### The relation between mother anxiety and child emotional distress

Moderation analyses were examined in a regression model constructed hierarchically, predicting child emotional distress. The results are presented in Table 2. The demographic characteristics did not contribute to the prediction; however, gender was marginally associated with child emotional distress, as girls presented higher levels of emotional distress. Higher maternal anxiety was related to higher levels of child emotional distress, whereas higher maternal self-efficacy was related to lower levels of child emotional distress. The interaction terms were entered in Step 2 and significantly contributed to the prediction. The entire model was significant, *F*(6, 123) = 5.90, *p* < 0.001.

**Table 2:** Moderators of the association between maternal anxiety and child emotional distress.

Child emotional distress			
Step	Predictor	*β*	Δ*R*^2^
Step 1	SES	−0.08	0.22***
	Child gender	−0.15+	
	Child age	−0.13	
	Maternal anxiety	0.30***	
	Maternal self-efficacy	−0.21***	
	War-related conversations	0.14	
Step 2	Maternal anxiety × war-related conversations	−19*	0.09***
	Maternal anxiety × Mother self-efficacy	−0.53***	
	*R*^2^ (Adjusted *R*^2^) = 0.31 (0.26)***		

* indicates *p* < 0.05. ** indicates *p* < 0.01, *** indicates *p* < 0.001, + indicates *p* < 0.10; SES, socio-economic status. War-related conversations were coded as 0 = absence of conversations and 1 = presence of conversations.

#### Simple slope analyses

**Figure 1:**
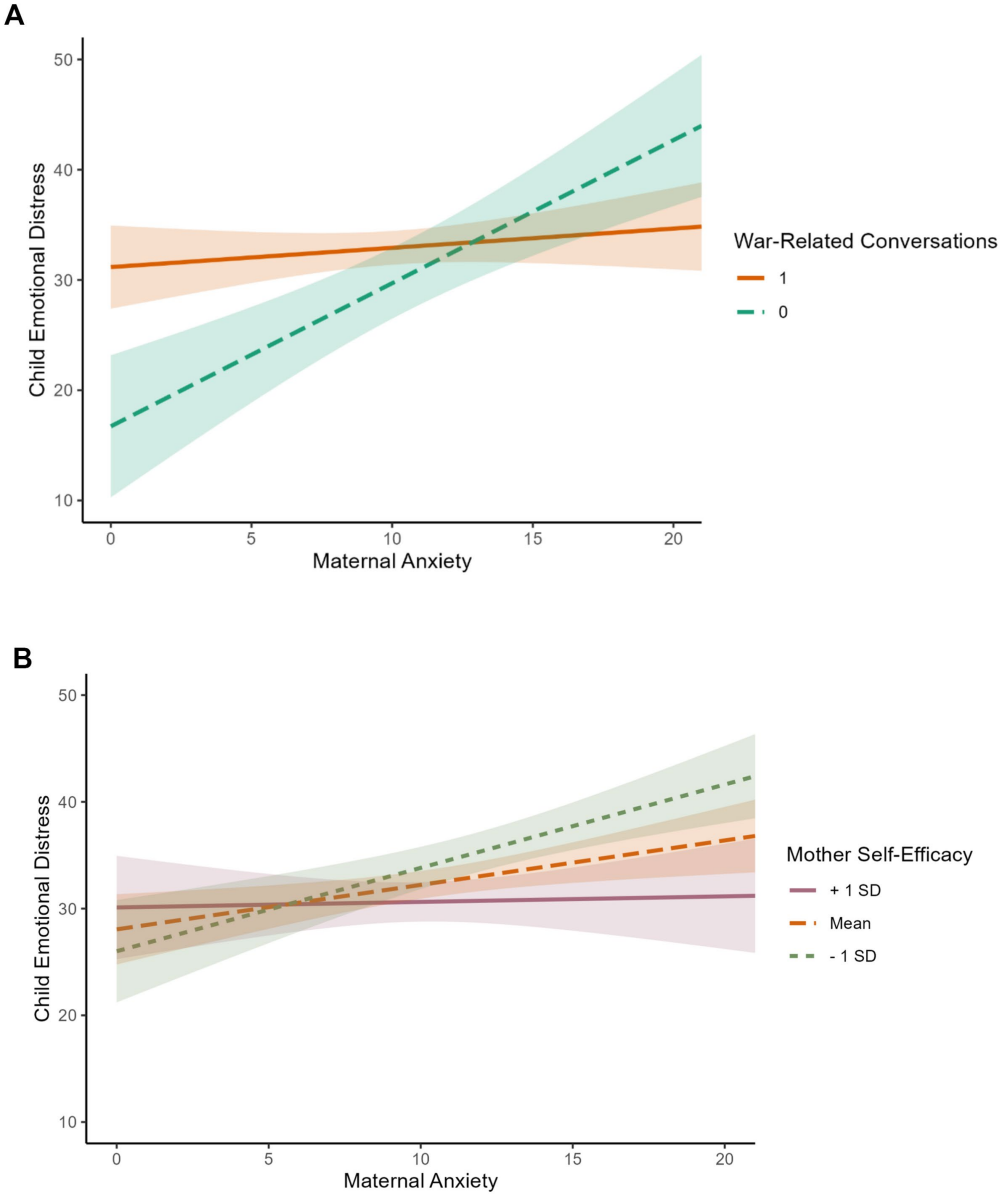
Simple slopes for the interaction between maternal anxiety and **(A)** war-related conversations and **(B)** maternal self-efficacy in predicting child emotional distress. Panel A presents the pattern of simple slopes for the interaction between maternal anxiety and war-related conversations in predicting child emotional distress. War-related conversations were assessed as a dichotomous variable (1 = presence; 0 = absence). Panel B presents the pattern of simple slopes for the interaction between maternal anxiety and maternal self-efficacy in predicting child emotional distress. The association between maternal anxiety and child emotional distress was estimated at low (−1 SD), average (Mean), and high (+1 SD) levels of maternal self-efficacy. Shaded areas represent 95% confidence intervals.

Simple slope analyses were conducted to examine the significant interaction between maternal anxiety and (1) war-related conversation, and (2) maternal self-efficacy. For the first interaction, the analysis indicated that the relation between maternal anxiety and child emotional distress was only significant among families who did not discuss the war (slope = 1.29, *t* = 4.83, *p* < 0.001, 95% CI [0.76; 1.83]). Among families who discussed the war, this relation was not significant (slope = 0.17, *t* = 1.01, *p* = 0.31, 95% CI [−0.16; 0.51]). The simple slopes patterns are presented in Figure 1, panel A. For the second interaction, the analysis indicated that the relation between maternal anxiety and child emotional distress was only significant among mothers with low or average levels of self-efficacy (slope = 1.01, *t* = 5.17, *p* < 0.001, 95% CI [0.68; 1.52], and slope = 0.73, *t* = 4.55, *p* < 0.001, 95% CI [0.42; 1.06], respectively). Among mothers with high levels of maternal self-efficacy, this relation was not significant (slope = 0.37, *t* = 1.65, *p* = 0.10, 95% CI [−0.08; 0.82]).

### Additional analysis

In this study, we included a wide age range of children (2–8 years). Child age was significantly correlated with maternal anxiety, maternal self-efficacy, and war-related conversations. For this reason, we controlled age in all previous analyses. Here, we further examined whether child age systematically moderated the previously observed effects, rather than treating age only as a covariate in the moderation analyses. To this end, we tested three-way interaction models using Model 3 of the PROCESS macro (Hayes et al., 2017). The three-way interaction between child age, maternal anxiety, and maternal self-efficacy was not significant, *β* = −0.01, Δ*R*^2^ = 0.00, *p* = 0.75; Similarly, the three-way interaction between child age, maternal anxiety, and war-related conversations did not reach statistical significance, *β* = −0.37, Δ*R*^2^ = 0.02, *p* = 0.07. These analyses suggest that the previously observed effects were consistent across the full age range of children.

## Discussion

The study examined Israeli families following the October 7th attacks and during the subsequent ‘Iron Swords’ war period, who were exposed to missile attacks and frequent air-raid sirens. The aim of the study was to examine the association between maternal anxiety and children’s emotional distress under conditions of heightened stress, and to identify moderating factors in this association. It was found that while maternal anxiety was generally associated with higher child emotional distress, this association was not significant among mothers with higher self-efficacy and among those who engaged in war related conversations with their children, suggesting that these factors functioned as protective moderators.

### The moderating role of maternal self-efficacy

Maternal self-efficacy showed both a main effect and a moderating effect, mitigating the association between high maternal anxiety and children’s emotional distress. The finding of the negative correlation between maternal self-efficacy and children’s emotional distress makes intuitive sense, as mothers who perceive themselves as more capable are likely better able to regulate their children’s emotional responses and help them navigate periods of stress. This is consistent with previous findings showing that mothers with a moderate and realistic sense of self-efficacy were most likely to respond sensitively to their infants’ distress (Leerkes and Crockenberg, 2002). Nevertheless, given the correlational design of the current study, it is also possible that children who exhibit fewer signs of distress contribute to higher maternal confidence and perceived parenting competence.

The association between maternal anxiety and child emotional distress was significant only among mothers with low to average levels of self-efficacy. For mothers with high self-efficacy, this relation was not significant. This suggests that maternal self-efficacy may function as a buffer that helps children cope with distress, even in the context of high maternal anxiety. For mothers with low to moderate self-efficacy, their perceived ability to help their children cope with challenges, such as the effects of war or other stressful events, may be more limited. However, it could also be that mothers with high self-efficacy may initially perceive less distress in their children, or that their children genuinely exhibit lower levels of anxiety, or that these mothers are less likely to recognize signs of distress in their children.

### The moderating role of parent–child communication about the war

The association between maternal anxiety and child emotional distress was moderated by war-related conversations. Importantly, there was no main effect of war-related conversations on child distress. Rather, the findings indicate that the relation between maternal anxiety and child emotional distress was evident only in families who did not discuss the war. When mothers reported engaging in conversations with their children about the war, maternal anxiety was not associated with child emotional distress. This pattern is particularly meaningful in cases of elevated maternal anxiety, which is otherwise related to higher child emotional distress. Research in the trauma literature suggests that parents may sometimes avoid discussing distressing events with their children; however, such avoidance may inadvertently exacerbate children’s stress (see systematic review of Afzal et al., 2023). Consistent with this view, our findings suggest that when mothers experience high levels of anxiety, engaging in conversations about the war, rather than avoiding them, may help children understand their mother’s anxiety and the surrounding stressful atmosphere, thereby attenuating the transmission of distress from mother to child.

A positive association was found between child age and whether mothers reported having war-related conversations with them. As children grow older, mothers tend to engage in more conversations with them on such topics. These findings reflect those of a systematic reviews assessing parent–child communication about potentially traumatic events (Afzal et al., 2023; Sloover et al., 2024). Parents were found to converse more with older children in a way they found to be appropriate developmentally. Older children receive more detailed and direct explanations of the stressful accounts. This may also reflect the children’s increasing curiosity, interest, and cognitive ability to ask questions and process complex events.

### Contextual factors related to maternal anxiety, maternal self-efficacy, and child emotional distress

Beyond the study’s original hypotheses, we also considered additional contextual factors, such as child gender, child age, and prior adverse life experiences, to provide additional context for interpreting the findings.

Consistent with previous findings (Bender et al., 2012), girls in our sample exhibited higher levels of emotional distress than boys. In addition, children’s age was positively associated with maternal anxiety and negatively associated with maternal self-efficacy. These findings may highlight the increasing challenges mothers face during extreme crises as their children grow older, as older children are more aware of and better able to understand their environment and the situation around them. Mothers of younger children often feel more in control of what their children are exposed to, such as what they watch, hear, and the kinds of conversations happening around them, this sense of control tends to diminish as children grow older (Fitzpatrick et al., 2023; Top, 2016). Older children are typically less under parental supervision and are more exposed to various influences through television, TikTok, social media, and peers (Poulain et al., 2023). They also tend to pick up on and understand more of what is being said around them, even when parents attempt to shield them from certain information. These factors may make it harder for mothers to feel that they can effectively protect their children, potentially increase their anxiety and reduce their sense of efficacy as parents. Importantly, however, the protective roles of maternal self-efficacy and war-related conversations were observed across the entire age range in the current study (i.e., 2–8 years old).

It is noteworthy that early exposure to potentially traumatic events or adverse life experiences was not associated with maternal anxiety. This finding is in contradiction with previous studies in this area (Bdier et al., 2023; Racine et al., 2021; Ring et al., 2024), which typically report such associations. A possible explanation for this discrepancy is the unique context of our study. Data was collected during a period of war, when maternal anxiety levels were elevated (i.e., 49% of the sample scored above the clinical threshold on the GAD-7). Under these conditions of high anxiety, the relative influence of past life experiences on current maternal anxiety may have been diminished, making associations that are usually observed less apparent. This finding may also be related to how prior adverse events were assessed. Specifically, in the current study previous adverse life events were evaluated using a single dichotomous (yes/no) item. This approach was chosen to avoid increasing participant burden given the length of the questionnaire. Consequently, our examination and interpretation of this variable are necessarily limited. Moreover, in such a complex state of stress, individuals with a history of adverse life experiences or trauma may actually demonstrate more adaptive responses to the current stressor (Morris and Rao, 2013). Interestingly, a history of mental health illness was also not associated with current maternal anxiety. Although the study was conducted with a general population sample, it is possible that those who chose to participate had sufficient psychological resources to engage with a study during such a sensitive time. Future research should aim to explore the nuanced effects of prior adverse life events and psychological vulnerabilities on maternal coping during periods of heightened stress. It is important to note that the primary objective of this study was not to examine the impact of these variables, but rather to investigate the complex relationship between maternal anxiety and the distress experienced by their children.

### Limitations

This study has several limitations. First, a major limitation of this study is its cross-sectional design. Although the study does not aim to make causal inferences, all variables were assessed at a single time point. As a result, the temporal ordering of the constructs cannot be determined, and the directionality of the observed associations remains unclear. It is therefore possible that the relationships identified are bidirectional or influenced by unmeasured third variables. Second, only mothers were included in the study, and were the sole reporters regarding their children’s experiences. While this reflects the reality that many fathers were actively involved in the war effort, leaving mothers to manage both their own anxieties and the day-to-day care of their children, the absence of fathers limits the scope of the findings. Fathers may express or perceive anxiety differently, and their inclusion could offer a more nuanced understanding of how parental stress relates to children’s emotional distress. Future research should aim to incorporate fathers’ voices as well. Third, our assessment of war-related conversations was limited, as included a single dichotomous question. While we asked mothers whether they explained the situation of the war and ongoing sirens to their children, we did not evaluate the quality or specific characteristics of these conversations, as might contribute to the comprehensive assessment of this aspect.

Fourth, the study is subject to potential sampling bias. Participation was voluntary and took place during an ongoing wartime period, which may have influenced who chose to take part. Individuals experiencing heightened emotional distress, increased caregiving demands or limited emotional or cognitive resources may therefore have been less likely to participate. In addition, the sample was relatively homogeneous in terms of socioeconomic characteristics, with the majority of participants classified as having average or above socioeconomic status.

Finally, the study was conducted during an ongoing war in a small country where the conflict had widespread impact. This context may limit the generalizability of the findings to other war-affected regions, particularly where the effects of conflict are less pervasive or differently experienced. At the same time, the psychological processes examined in the current study, namely the transmission of maternal anxiety to child emotional distress, and the potential buffering roles of maternal self-efficacy and war-related communication are grounded in broader theoretical frameworks that are not specific to the Israeli context. Therefore, while caution is warranted in generalizing the magnitude or specific expression of the effects, the main findings may be relevant to other populations facing chronic stress, armed conflict, or large-scale collective threat. Future research conducted in diverse cultural and conflict settings is needed to further examine the robustness and boundary conditions of these findings.

## Conclusion

The current study highlights the importance of understanding the relations between maternal anxiety and child emotional distress. The findings suggest that higher maternal self-efficacy and engaging in conversations with the child about war-related events may serve as protective buffers against the negative effects of high maternal anxiety. Supporting parents in increasing their sense of efficacy may not only help them cope during times of extreme stress, such as war, but also enhance the resilience of both parents and children.

## Data Availability

The data that support the findings of this study are available from the corresponding author upon reasonable request.
